# Antibiotic Elution from Hip and Knee Acrylic Bone Cement Spacers: A Systematic Review

**DOI:** 10.1155/2017/4657874

**Published:** 2017-06-05

**Authors:** Konstantinos Anagnostakos, Christof Meyer

**Affiliations:** Zentrum für Orthopädie und Unfallchirurgie, Klinikum Saarbrücken, Saarbrücken, Germany

## Abstract

Knowledge about the elution from antibiotic-loaded cement spacers is an indispensable premise for guarantee of clinical success. A systematic literature search was performed through PubMed. Search terms were “antibiotic elution” and “antibiotic release” in combination with “spacer,” “hip spacer,” and “knee spacer,” respectively. A total of 11 studies could be identified. Seven studies reported on the release of antibiotics after spacer implantation, three studies at spacer removal, and one study on both time points. Seven studies reported on hip spacers, one study on knee spacers, and three studies on both. In eight studies, custom-made spacers have been implanted and in three prefabricated ones. In the majority of the studies, the cement has been loaded with an antibiotic combination, mostly consisting of aminoglycoside (either gentamicin or tobramycin) and vancomycin. Measured concentrations exceeded the minimal inhibitory concentration of the particular pathogen organisms in each case. However, large discrepancies were observed with regard to the height of the antibiotic concentration depending on the antibiotic combination and the antibiotic ratio used. Current literature data indicate a sufficient elution of antibiotics after spacer implantation and at spacer removal, respectively. Future studies are required to optimize the local antibiotic therapy at the site of spacer implantation.

## 1. Introduction

Periprosthetic joint infections pose a great challenge for the orthopaedic surgeon. At the site of late infections, the implantation of antibiotic-loaded cement spacers is the treatment of choice in Europe and North America with success rates exceeding 90% [1, 2].

Cement spacers have several advantages such as high local antibiotic therapy, preservation of joint mobility in case of articulating ones, less formation of scar tissue, and ease of prosthesis reimplantation [1, 2]. The ideal spacer should possess sufficient pharmacokinetic properties over a prolonged time period in order to eradicate the infection and prevent the emergence of new, multiresistant bacteria strains, if any should have survived after spacer implantation.

The release of antibiotics from cement spacers has been well studied in vitro [3–5]. However, these results cannot be directly transferred to clinical practice. Differences in the cement used and its antibiotic impregnation, addition of one or combination of two or more antibiotics, the amount and/or ratio of the incorporated antibiotic(s), length of spacer implantation, spacer geometry and surface, and spacer articulation are only some of the factors that might have a possible influence on the pharmacokinetic properties in vivo.

The aim of the present work is to summarize the current knowledge about the elution of antibiotics from hip and knee spacers in vivo and distinguish between the release kinetics after spacer implantation and at spacer removal, respectively.

## 2. Materials and Methods

### 2.1. Inclusion of Studies

**Figure 1:**
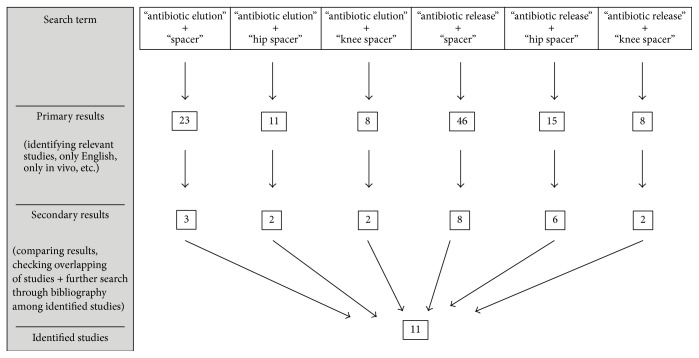
Literature search for identification of studies about antibiotic elution from acrylic bone cement hip and knee spacers in vivo.

A literature search was performed through PubMed until November 2016 (Figure 1). Search terms were “antibiotic elution” and “antibiotic release” in combination with “spacer”, “hip spacer”, and “knee spacer”, respectively. Only English studies and those solely reporting about the release of antibiotics from acrylic bone cement hip and knee spacers in vivo were included. In vitro studies, reviews, and case reports were excluded. Among the primarily identified studies, a search was carried out through the bibliography of each article for further identification of relevant studies. All publications were analyzed with regard to joint localization, number of spacer implantations, cement used, antibiotic impregnation, type of spacer, time of measurement of the antibiotic elution (after spacer implantation versus at spacer removal), and pharmacokinetic findings.

## 3. Results

A total of 11 studies [6–16] could be identified (Table 1). Seven studies reported on the release of antibiotics after spacer implantation [6–8, 11–13, 16], three studies at spacer removal [9, 14, 15], and one study on both time points [10]. Seven studies reported on hip spacers [6, 9–13, 16], one study on knee spacers [15], and three studies on both [7, 8, 14] (Table 1). In eight studies, custom-made spacers have been implanted and in three prefabricated ones. Only one study investigated the properties of cement spacers impregnated with a single antibiotic [12]. In all other studies, the cement has been loaded with an antibiotic combination, mostly consisting of an aminoglycoside (either gentamicin or tobramycin) and vancomycin. In all studies, the incorporated antibiotics were in powder form except for one [11]. All data about the production and antibiotic impregnation details of hip and knee spacers are summarized in Table 1.

### 3.1. Antibiotic Elution: After Spacer Implantation

All studies determined the local antibiotic concentrations in the joint fluid collected in the redon drain. The time period of measurement varied between the first 24 postoperative hours and seven postoperative days after spacer implantation. In the studies with a longer measurement period, the antibiotic elution demonstrated a biphasic profile, consisting of initial high concentrations, which then constantly decreased over time. The highest values determined were observed in a study where vancomycin and aztreonam were incorporated into 40 g bone cement with initial values exceeding 1,000 *µ*g/ml (impregnation dose 3 g vancomycin + 4 g aztreonam/40 g cement; average amount of cement/spacer 86.7 ± 10.3 g) [10]. At the site of a gentamicin-vancomycin combination, some large discrepancies with regard to height of the measured concentrations could be seen among the studies. Anagnostakos et al. determined maximum concentrations of gentamicin and vancomycin at 39 and 72 *µ*g/ml, respectively, when 1 g gentamicin and 4 g vancomycin (both powder) were impregnated into 80 g cement for spacer production. Hsieh et al. reported mean local levels of gentamicin at 58.3 mg/l and of vancomycin at 485.5 mg/l on day 1 when liquid gentamicin was combined with vancomycin powder (impregnation dose 480 mg gentamicin + 3 g vancomycin/40 g cement; average amount of cement/spacer 72.2 ± 11.4 g) [11]. All initially determined concentrations in all studies were beyond the minimal inhibitory concentrations of the causative bacteria. All data about the antibiotic elution after spacer implantation is summarized in Table 2.

### 3.2. Antibiotic Elution: At Spacer Removal

Three studies determined the antibiotic concentrations in the local joint fluid found at spacer removal, whereas one study measured them in the local tissue. The length of spacer implantation varied from six weeks to several months. All measured concentrations were above the minimal inhibitory concentration of the particular causative organism in each study despite an apparent trend toward decreasing levels over time seen in two studies [11, 14]. At the site of hip spacers, no significant differences could be found between the levels associated with acetabular cup and those with spacer stem implantation [9]. In another study, no significant differences were observed between hip and knee spacers [14]. When tobramycin was combined with vancomycin, tobramycin itself and the increase of its incorporated dose had an influence on the elution kinetics of both antibiotics but not vice versa [14]. All data about the antibiotic elution at spacer removal is summarized in Table 3.

## 4. Discussion

Knowledge about the antibiotic elution from cement spacers is an indispensable premise for guarantee of infection eradication in the management of periprosthetic hip and knee infections. The present work tried to systematically review the current literature about this topic and differ between the pharmacokinetic properties after spacer implantation and at spacer removal.

The evaluation of the efficacy of spacers with different antibacterial loads based on published reports is difficult [17]. As aforementioned, it is apparent that numerous factors might theoretically have an influence on the release kinetics from bone cement in vivo. Generally, the type and ratio of antibiotics, the quantity of antibiotic(s), the type and porosity of cement, the surface characteristics, the way the cement is prepared, and the environmental circumstances are accepted to be factors with a possible affection on the antibiotic release from bone cement [18]. Therefore, the interpretation of in vivo studies about the elution of antibiotics from cement spacers has some great differences compared with in vitro studies. The majority of the knowledge about elution kinetics from antibiotic-loaded bone cement origins from studies that investigated cement device other than spacers such as disks [19–22]. Since the release of antibiotics from bone cement is a surface-dependent phenomenon [23], it is questionable to what effect these in vitro observations also account for hip and knee spacers that have a different geometry and surface. Moreover, the amount and frequency of fluid exchange around bone cement in vitro do not fully represent the vascular supply nor the resulting antibiotic diffusion to tissue and hence the in vivo circumstances which certainly cause a different wash-out phenomenon of antibiotics from the cement.

Based on these considerations, it is essential to study and understand the pharmacokinetic properties of cement spacers in vivo. Here, specific factors such as place and length of measurement should be critically evaluated. In the majority of the cases among the identified studies, the elution of hip spacers was studied. After spacer implantation, the determination of antibiotic concentrations occurred in joint fluids collected from the drains during the first postoperative days. However, these concentrations are not fully representative for the whole pharmacokinetic properties from the spacers but mostly for the intra-articular spacer part. Especially at the site of hip spacers, this accounts only for the pharmacokinetic properties of either the spacer head alone or the spacer head in combination with a spacer cup. The antibiotic release from the spacer stem in the femoral cavity cannot be determined in the joint fluid. Therefore, the measured concentrations represent only a part of the true antibiotic elution in vivo, and this has to be born in mind.

Moreover, the elution properties might depend on the fluid amount that washes the antibiotics out from the bone cement. It could be possible that the initially high antibiotic elution causes a quick saturation of the surrounding tissue. The diffusion gradient that supports the antibiotic release at the beginning is decreased while the tissue saturation is increasing. This could lead to a severe time-dependent reduction of the antibiotic release from spacers [13]. In contrast to that finding, the diffusion gradient in vitro is permanently high. Since in most studies the culture medium is changed daily, the permanently existing differences between spacer surface and culture medium are causing a new, high, and longer lasting antibiotic elution. Unfortunately, the fluid amount in the drains was not always stated in the identified studies.

By a constant decrease of the fluid amount during the early postoperative period, the antibiotic release shows then a normal decrease. If the spacers would be again exposed to “fresh” fluids, the elution of antibiotics could be restarted. This phenomenon could be observed in the studies of Bertazzoni Minelli et al. [24] and Kelm et al. [13]. Both studies investigated the residual pharmacokinetic and associated antimicrobial properties of spacers after their explantation in vitro. 0.05–0.4% gentamicin and 0.8–3.3% vancomycin of the initial amount present were released over a time period of 10 days in the first study [24], indicating that sufficient antibiotic release can persist over several months. Kelm et al. reported similar elution values of gentamicin and vancomycin, and their spacers demonstrated sufficient antimicrobial properties for at least 14 days in vitro independent of their implantation period [13].

The type of antibiotics themselves used for cement impregnation plays also an important role with regard to release kinetics. In the present evaluation, all studies except for one solely used antibiotics in powder form. Antibiotics in powder form have a lower impact on the mechanical properties of bone cement at a ratio of up to 10% [25], whereas antibiotics in liquid form enhance the pharmacokinetic properties while having a negative impact on the mechanical stability [26]. Adding liquid antibiotics reduces the compressive strength of bone cement by 49% and the tensile strength by 46% [27]. Cement spacers are thought to be only interim prostheses so that in the majority of the cases mechanical complications such as spacer fractures might be avoided when liquid antibiotics are used and if the patient is able to put non-weight-bearing on the operated leg until the prosthesis reimplantation. However, in some cases, a prosthesis reimplantation cannot be performed for various reasons, and the patients are left with their spacer in situ [28–30]. Therefore, it is questionable whether the impregnation of bone cement with liquid antibiotics might be advisable and safe, even if the antibiotic elution is hereby enhanced.

The choices of bone cement and incorporated antibiotics are probably the two most important factors at the site of cement spacers. Palacos® has been regarded to be the cement type with the best pharmacokinetic properties for many years [31, 32]. However, in the past years, several studies have indicated that other bone cements have at least equally good or even superior elution properties as Palacos has [3, 19, 33, 34]. Bitsch et al. investigated in vitro the release of several antibiotics from a new cement, especially designed for spacer production, and could determine a significantly higher and prolonged antibiotic elution for a period of up to 50 days [3]. Neut et al. demonstrated for Palamed the best elution kinetics among six tested gentamicin-loaded bone cements [33]. Similar in vitro observations were also made by van de Belt and colleagues [34]. Cerretani et al. demonstrated that when vancomycin was incorporated alone into PMMA, the highest elution rates occur from CMW 1 compared to Simplex® and Palacos; however, when combined with imipenem-cilastatin Palacos and Simplex have superior pharmacokinetic properties [19]. These discrepancies among the elution kinetics of different bone cement types might pose a possible explanation for the very high elution of antibiotics observed in one identified study, where Simplex cement was used compared to the other studies (Tables 1 and 2).

Although it is known that commercially available antibiotic-impregnated bone cements have superior elution properties than those with a manual impregnation of an antibiotic due to the more homogenous distribution of the incorporated antibiotics in the cement powder, orthopaedic surgeons frequently need to add other antimicrobial agents to the bone cement of spacers due to the sensitivity profile of the causative pathogen organism. Therefore, knowledge about the synergism between the incorporated antibiotics is important for the clinical performance. Despite the fact that the precise synergism mechanism is not completely understood, it seems that this mechanism can be attributed to the so-called passive opportunism [22]. The second antibiotic appears to act as a soluble additive increasing porosity and thereby enhancing the elution of the first antibiotic or both. When an aminoglycoside (gentamicin or tobramycin) has been combined with vancomycin, in vitro studies have demonstrated a synergistic effect for one [35] or both agents [22] which is maintained in cement when both drugs are released in active form at site of infection. Apparently the amount of synergism might also depend on the ratio of the antibiotics which might also explain the partly large discrepancies with regard to height of antibiotic concentrations released from cement spacers as shown in the present work (Tables 2 and 3).

Due to the decrease of the antibiotic elution from spacers over time, some concerns have been expressed in the past years about the possible growth of bacteria on spacers, hence leading to clinical infection persistence. The present literature shows some partly contradictory results about this topic. Some studies determined the bacteria growth on spacers after their explantation by sonication and could show a bacteria growth on spacers to different percentages, respectively [36–38]. In some cases, the spacers were not loaded with antibiotics which could explain the bacteria growth [38]. However, not every case was associated with a clinical infection persistence [36–38]. Another study investigated this phenomenon by scanning electron microscopy and confocal scanning light microscopy and could not detect any biofilm formation on the spacers [39]. Hence, the theoretical possibility of bacteria growth on spacers is present but cannot be surely supported by hard scientific data.

Despite numerous studies about hip and knee spacers, several topics still remain unclear with regard to the antibiotic impregnation. None of the identified studies investigated whether custom-made or prefabricated spacers release more antibiotic amounts or higher concentrations, over a prolonged period. Moreover, the properties of antibiotic combinations other than the combination of an aminoglycoside and a glycopeptide are not known. At present, some antibiotic combinations are potential because the choice is limited by safety issue (fluoroquinolones and bone, beta-lactam drugs, and sensitivity/allergy), stability, and compatibility with cement. Last but not least, the ideal ratio for impregnation of bone cement in vivo is also unclear.

## 5. Conclusion

The management of late periprosthetic infections by means of a spacer implantation is an established method. Current literature data indicate a sufficient elution of antibiotics after spacer implantation and at spacer removal, respectively. However, some large discrepancies with regard to height and length of the sufficient antibiotic release are evident among the identified studies. Future studies are required to optimize the local antibiotic therapy at the site of spacer implantation.

## Figures and Tables

**Table 1:** Production and antibiotic impregnation details of hip and knee spacers.

Study	Joint	Number of spacer implantations	Cement used	Spacer type	Antibiotic impregnation and type
Anagnostakos et al. [6]	Hip	17	Palacos	Custom-made	0.5 g G + 2 g V/40 g cement powder

Balato et al. [7]	10x hip 8x knee	18	Palacos	Custom-made	1 g G + 1 g C powder

Bertazzoni Minelli et al. [8]	5x hip 6x knee	11	Cemex®	Prefabricated^*∗*^	1.9% G, 1.9% G + 1.25% V, 1.9% G + 1.9% V, 1.9% G + 2.5% V, 1.9% G + 5% V powder

Fink et al. [9]	Hip	14	Palacos	Custom-made	7 × 1 g G + 1 g C/40 g cement, 7 x 1 g G + 1 g C + 2 g V/40 g cement powder

Hsieh et al. [10]	Hip	46	Simplex	Custom-made	4 g V + 4 g A/40 g cement powder

Hsieh et al. [11]	Hip	42	Simplex	Custom-made	~300 mg G + 3 g V/40 g cement liquid G + powder V

Isiklar et al. [12]	Hip	10	n.r.	Custom-made	2 g V/40 g cement powder

Kelm et al. [13]	Hip	10	Palacos	Custom-made	0.5 g G + 2 g V/40 g cement

Masri et al. [14]	34x hip 15x knee	49	37x Palacos 12x Simplex	Custom-made	1.2–4.8 g T + 1-2 g V/40 g cement powder

Mutimer et al. [15]	Knee	12	Cemex	Prefabricated	Gentamicin powder

Regis et al. [16]	Hip	7	Cemex	Prefabricated^*∗∗*^	2.5% G + 150–170 mg V powder

V: vancomycin; G: gentamicin; C: clindamycin; A: aztreonam; T: tobramycin; *∗*: in 9/11 cases additional impregnation by drilling in the spacer and filling with vancomycin-loaded cement; *∗∗*: in all cases additional impregnation by drilling in the spacer and filling with vancomycin-loaded cement; n.r.: not reported.

**Table 2:** Findings about the antibiotic elution from hip and knee spacers after spacer implantation in vivo.

Study	Place of measurement	Time period of measurement	Pharmacokinetic findings	Infection control
Anagnostakos et al. [6]	Joint fluid	First 7 postop. days	[G]_MAX_ 39 *µ*g/ml, [V]_MAX_ 72 *µ*g/ml on day 1 [G]_MIN_ 1.9 *µ*g/ml, [V]_MIN_ 6.6 *µ*g/ml on day 7 Between days 5 and 7 intermittent increase in the elution of both antibiotics	n.r.

Balato et al. [7]	Joint fluid	First 48 postop. hours	[G]_MAX_ 53.9 *µ*g/ml in the hip group, [G]_MAX_ 44.1 *µ*g/ml in the knee group Mean [G] significantly higher in the hip than in the knee group (30.61 ± 19.47 *µ*g/ml versus 17.43 ± 13.63 *µ*g/ml) one hour after implantation	100%

Bertazzoni Minelli et al. [8]	Joint fluid	First 24 postop. hours	[G]_MAX_ 88 *µ*g/ml in the hip group, [G]_MAX_ 110 *µ*g/ml in the knee group [V]_MAX_ 28.8 *µ*g/ml in the hip group, [V]_MAX_ 158.9 *µ*g/ml in the knee group	100%

Hsieh et al. [10]	Joint fluid	First 7 postop. days	Mean [V]_MAX_ 1,538 *µ*g/ml, mean [A]_MAX_ 1,003.5 *µ*g/ml on day 1 Mean [V]_MIN_ 571.9 *µ*g/ml, mean [A]_MIN_ 313.6 *µ*g/ml on day 7	97.8%

Hsieh et al. [11]	Joint fluid	First 7 postop. days	Mean [G]_MAX_ 58.3 *µ*g/ml, mean [V]_MAX_ 485.5 *µ*g/ml on day 1 Mean [G]_MIN_ 14.6 *µ*g/ml, mean [V]_MIN_ 76.1 *µ*g/ml on day 7	95.2%

Isiklar et al. [12]	Joint fluid	First 24 postop. hours	Mean [V] 57 [32–81] *µ*g/ml	100%

Kelm et al. [13]	Joint fluid	First 7 postop. days	Vancomycin quantitatively higher than gentamicin extrapolated concentration-time curves showed power functions, so that subtherapeutic antibiotic levels can be expected for vancomycin on the 17th postop. day and for gentamicin on the 14th postop. day	n.r.

Regis et al. [16]	Joint fluid	First 24 postop. hours	[G] ranged between 15 and 90 *µ*g/ml; [V] ranged between 13.8 and 40 *µ*g/ml	n.r.

G: gentamicin; V: vancomycin; A: aztreonam; MAX: maximum; MIN: minimum; n.r.: not reported.

**Table 3:** Findings about the antibiotic elution from hip and knee spacers at spacer removal.

Study	Place of measurement	Length of spacer implantation	Pharmacokinetic findings	Infection control
Fink et al. [9]	Local tissue	Six weeks	[G]_MAX_ 50.93 *µ*g/g, [V]_MAX_ 177.24 *µ*g/g, [C]_MAX_ 322.29 *µ*g/g No differences in [G] and [C] regardless of whether V has been added to cement No differences between levels associated with acetabular cup and those with spacer stem	n.r.

Hsieh et al. [10]	Joint fluid	Mean 107 [32–156] days	All [V] and [A] above the MIC despite an apparent trend toward decreasing levels over time	97.8%

Masri et al. [14]	Joint fluid	Mean 118 [42–340] days	No significant differences between hip and knee spacers Highest [T] and [V] when at least 3.6 g T was impregnated Significant increase when the dose of T was increased from at most 2.4 g to at least 3.6 g per cement package V has no significant influence on [T] Increase of the V dose from 1 to 1.5–2 g V per cement package with no significant influence on [T] or [V] Apparent trend toward decreasing levels over time	n.r.

Mutimer et al. [15]	Joint fluid	Median 99 [63–274] days	Median [G] 0.46 [0.24–2.36] *µ*g/ml	100%

G: gentamicin; V: vancomycin; C: clindamycin; A: aztreonam; T: tobramycin; MAX: maximum; MIN: minimum; MIC: minimal inhibitory concentration; n.r.: not reported.

## References

[1] Anagnostakos K., Fürst O., Kelm J. (2006). Antibiotic-impregnated PMMA hip spacers: current status. *Acta Orthopaedica*.

[2] Cui Q., Mihalko W. M., Shields J. S., Ries M., Saleh K. J. (2007). Antibiotic-impregnated cement spacers for the treatment of infection associated with total hip or knee arthroplasty. *Journal of Bone and Joint Surgery - Series A*.

[3] Bitsch R. G., Kretzer J. P., Vogt S., Büchner H., Thomsen M. N., Lehner B. (2015). Increased antibiotic release and equivalent biomechanics of a spacer cement without hard radio contrast agents. *Diagnostic Microbiology and Infectious Disease*.

[4] Goltzer O., McLaren A., Overstreet D., Galli C., McLemore R. (2015). Antimicrobial release from prefabricated spacers is variable and the dose is low. *Clinical Orthopaedics and Related Research*.

[5] Stevens C. M., Tetsworth K. D., Calhoun J. H., Mader J. T. (2005). An articulated antibiotic spacer used for infected total knee arthroplasty: A comparative in vitro elution study of Simplex® and Palacos® bone cements. *Journal of Orthopaedic Research*.

[6] Anagnostakos K., Wilmes P., Schmitt E., Kelm J. (2009). Elution of gentamicin and vancomycin from polymethylmethacrylate beads and hip spacers in vivo. *Acta Orthopaedica*.

[7] Balato G., Ascione T., Rosa D. (2015). Release of gentamicin from cement spacers in two-stage procedures for hip and knee prosthetic infection: an in vivo pharmacokinetic study with clinical follow-up. *Journal of Biological Regulators & Homeostatic Agents*.

[8] Bertazzoni Minelli E., Benini A., Samaila E., Bondi M., Magnan B. (2015). Antimicrobial activity of gentamicin and vancomycin combination in joint fluids after antibiotic-loaded cement spacer implantation in two-stage revision surgery. *Journal of Chemotherapy*.

[9] Fink B., Vogt S., Reinsch M., Büchner H. (2011). Sufficient release of antibiotic by a spacer 6 weeks after implantation in two-stage revision of infected hip prostheses. *Clinical Orthopaedics and Related Research*.

[10] Hsieh P.-H., Chang Y.-H., Ueng S. W. N., Shih C.-H. (2006). High concentration and bioactivity of vancomycin and aztreonam eluted from simplex cement spacers in two-stage revision of infected hip implants: a study of 46 patients at an average follow-up of 107 days. *Journal of Orthopaedic Research*.

[11] Hsieh P.-H., Huang K.-C., Tai C.-L. (2009). Liquid gentamicin in bone cement spacers: in vivo antibiotic release and systemic safety in two-stage revision of infected hip arthroplasty. *Journal of Trauma—Injury, Infection and Critical Care*.

[12] Isiklar Z. U., Demirörs H., Akpinar S., Tandogan R. N., Alparslan M. (1999). Two-stage treatment of chronic staphylococcal orthopaedic implant- related infections using vancomycin impregnated PMMA spacer and rifampin containing antibiotic protocol. *Bulletin: Hospital for Joint Diseases*.

[13] Kelm J., Regitz T., Schmitt E., Jung W., Anagnostakos K. (2006). In vivo and in vitro studies of antibiotic release from and bacterial growth inhibition by antibiotic-impregnated polymethylmethacrylate hip spacers. *Antimicrobial Agents and Chemotherapy*.

[14] Masri B. A., Duncan C. P., Beauchamp C. P. (1998). Long-term elution of antibiotics from bone-cement: an in vivo study using the prosthesis of antibiotic-loaded acrylic cement (PROSTALAC) system. *The Journal of Arthroplasty*.

[15] Mutimer J., Gillespie G., Lovering A. M., Porteous A. J. (2009). Measurements of in vivo intra-articular gentamicin levels from antibiotic loaded articulating spacers in revision total knee replacement. *Knee*.

[16] Regis D., Sandri A., Samaila E., Benini A., Bondi M., Magnan B. (2013). Release of gentamicin and vancomycin from preformed spacers in infected total hip arthroplasties: measurement of concentrations and inhibitory activity in patients' drainage fluids and serum. *The Scientific World Journal*.

[17] Iarikov D., Demian H., Rubin D., Alexander J., Nambiar S. (2012). Choice and doses of antibacterial agents for cement spacers in treatment of prosthetic joint infections: Review of published studies. *Clinical Infectious Diseases*.

[18] Anagnostakos K., Kelm J. (2009). Enhancement of antibiotic elution from acrylic bone cement. *Journal of Biomedical Materials Research Part B: Applied Biomaterials*.

[19] Cerretani D., Giorgi G., Fornara P. (2002). The in vitro elution characteristics of vancomycin combined with imipenem-cilastatin in acrylic bone-cements: a pharmacokinetic study. *The Journal of Arthroplasty*.

[20] Scott C. P., Higham P. A. (2003). Antibiotic bone cement for the treatment of pseudomonas aeruginosa in joint arthroplasty: comparison of tobramycin and gentamicin-loaded cements. *Journal of Biomedical Materials Research*.

[21] Streuli J. C., Exner G. U., Reize C. L., Merkofer C., Scott C. P., Zbinden R. (2006). In vitro inhibition of coagulase-negative staphylococci by vancomycin/aminoglycoside-loaded cement spacers. *Infection*.

[22] Penner M. J., Masri B. A., Duncan C. P. (1996). Elution characteristics of vancomycin and tobramycin combined in acrylic bone-cement. *The Journal of Arthroplasty*.

[23] Holtom P. D., Warren C. A., Greene N. W. (1998). Relation of surface area to in vitro elution characteristics of vancomycin-impregnated polymethylmethacrylate spacers. *American Journal of Orthopedics*.

[24] Bertazzoni Minelli E., Benini A., Magnan B., Bartolozzi P. (2004). Release of gentamicin and vancomycin from temporary human hip spacers in two-stage revision of infected arthroplasty. *The Journal of Antimicrobial Chemotherapy*.

[25] Lautenschlager E. P., Jacobs J. J., Marshall G. W., Meyer P. R. (1976). Mechanical properties of bone cements containing large doses of antibiotic powders. *Journal of Biomedical Materials Research*.

[26] Hsieh P.-H., Tai C.-L., Lee P.-C., Chang Y.-H. (2009). Liquid gentamicin and vancomycin in bone cement. A potentially more cost-effective regimen. *Journal of Arthroplasty*.

[27] Kuechle D. K., Landon G. C., Musher D. M., Noble P. C. (1991). Elution of vancomycin, daptomycin, and amikacin from acrylic bone cement. *Clinical Orthopaedics and Related Research*.

[28] Amouyel T., Brunschweiler B., Freychet B., Lautridou C., Rosset P., Massin P. (2015). No improvement in the post-TKA infection prognosis when the implant is not reimplanted: retrospective multicentre study of 72 cases. *Orthopaedics and Traumatology: Surgery and Research*.

[29] Choi H.-R., Freiberg A. A., Malchau H., Rubash H. E., Kwon Y.-M. (2014). The fate of unplanned retention of prosthetic articulating spacers for infected total hip and total knee arthroplasty. *Journal of Arthroplasty*.

[30] Lee W., Hwang D., Kang C., Shin B., Zheng L. (2017). Usefulness of prosthesis made of antibiotic-loaded acrylic cement as an alternative implant in older patients with medical problems and periprosthetic hip infections: a 2- to 10-year follow-up study. *The Journal of Arthroplasty*.

[31] Wahlig H. (1987). Kinetics of the liberation of antibiotics from bone cements results of comparative studies in vitro and in vivo. *Aktuelle Probleme in Chirurgie und Orthopädie*.

[32] Penner M. J., Duncan C. P., Masri B. A. (1999). The in vitro elution characteristics of antibiotic-loaded CMW and Palacos-R bone cements. *Journal of Arthroplasty*.

[33] Neut D., van de Belt H., van Horn J. R., van der Mei H. C., Busscher H. J. (2003). The effect of mixing on gentamicin release from polymethylmethacrylate bone cements. *Acta Orthopaedica Scandinavica*.

[34] van de Belt H., Neut D., Uges D. R. A. (2000). Surface roughness, porosity and wettability of gentamicin-loaded bone cements and their antibiotic release. *Biomaterials*.

[35] Klekamp J., Dawson J. M., Haas D. W., DeBoer D., Christie M. (1999). The use of vancomycin and tobramycin in acrylic bone cement: biomechanical effects and elution kinetics for use in joint arthroplasty. *The Journal of Arthroplasty*.

[36] Nelson C. L., Jones R. B., Wingert N. C., Foltzer M., Bowen T. R. (2014). Sonication of antibiotic spacers predicts failure during two-stage revision for prosthetic knee and hip infections. *Clinical Orthopaedics and Related Research*.

[37] Schmolders J., Hischebeth G. T. R., Friedrich M. J. (2014). Evidence of MRSE on a gentamicin and vancomycin impregnated polymethyl-methacrylate (PMMA) bone cement spacer after two-stage exchange arthroplasty due to periprosthetic joint infection of the knee. *BMC Infectious Diseases*.

[38] Sorlí L., Puig L., Torres-Claramunt R. (2012). The relationship between microbiology results in the second of a two-stage exchange procedure using cement spacers and the outcome after revision total joint replacement for infection: the use of sonication to aid bacteriological analysis. *Journal of Bone and Joint Surgery - Series B*.

[39] Griffin J. W., Guillot S. J., Redick J. A., Browne J. A. (2012). Removed antibiotic-impregnated cement spacers in two-stage revision joint arthroplasty do not show biofilm formation in vivo. *Journal of Arthroplasty*.

